# Gene Polymorphisms of *FABP2*, *ADIPOQ* and *ANP* and Risk of Hypertriglyceridemia and Metabolic Syndrome in Afro-Caribbeans

**DOI:** 10.1371/journal.pone.0163421

**Published:** 2016-09-29

**Authors:** Laurent Larifla, Christine Rambhojan, Marie-Odile Joannes, Suliya Maimaitiming-Madani, Jean-Paul Donnet, Thérèse Marianne-Pépin, Roger Chout, Ronan Roussel, Lydia Foucan

**Affiliations:** 1 Equipe de recherche sur le risque cardiométabolique ECM/LAMIA EA4540, Université des Antilles, Guadeloupe, France; 2 Service de cardiologie, Centre Hospitalier Universitaire de Pointe-à-Pitre, Guadeloupe, France; 3 Novo Nordisk, Paris, France; 4 Service d’endocrinologie, diabétologie et nutrition, Assistance Publique des Hôpitaux de Paris, Hôpital Bichat, DHU FIRE, Paris, France; Wake Forest School of Medicine, UNITED STATES

## Abstract

**Objectives:**

The metabolic syndrome (MetS) is a cluster of metabolic abnormalities and cardiovascular risk factors that are highly heritable and polygenic. We investigated the association of allelic variants of three candidate genes, rs1799883-*FABP2*, rs1501299-*ADIPOQ* and rs5065-*ANP* with MetS and its components, individually and in combination, using a genetic risk score.

**Methods:**

A cross-sectional study was conducted in 462 Afro-Caribbeans subjects without cardiovascular complications or lipid-lowering medications. Cardiovascular risk factors and MetS components (NCEP-ATPIII criteria) were recorded. The 3 SNPs were genotyped. The genetic risk score was calculated by summing the number of risk alleles at each locus. Logistic regressions were used.

**Results:**

Fifty-eight participants (12.6%) were diabetics and 116 (25.1%) had a MetS. In a dominant model, rs1799883 was associated with hypertriglyceridemia (OR 2.22; *P* = 0.014) and hypertriglyceridemic waist (HTGW), (*P* = 0.014) but not significantly with overweight (*P* = 0.049), abdominal obesity (*P* = 0.033) and MetS (*P* = 0.068). In a dominant model, the OR of MetS and HTGW for rs1501299 were 1.80 (*P* = 0.028) and 2.19 (*P* = 0.040) respectively. In a recessive model, the OR of hypertriglyceridemia for rs5065 was 1.94 (*P* = 0.075). The genetic risk score was significantly associated with MetS. Subjects carrying 4–5 risk alleles (18.8%) had a nearly 2.5-fold-increased risk of MetS compared to those carrying 0–1 risk allele (24.3%): OR 2.31; *P* = 0.025.

**Conclusions:**

This study supports the association of *FABP2*, *ANP* and *ADIPOQ* gene variants with MetS or its components in Afro-Caribbeans and suggests a cumulative genetic influence of theses variants on this syndrome and a potential effect on lipid metabolism.

## Introduction

The metabolic syndrome (MetS) is a set of metabolic and cardiovascular risk factors associated with an increased prevalence of diabetes and cardiovascular diseases. The underlying cause of MetS is still unclear but, central obesity, adipose tissue dysregulation and insulin-resistance are considered as key contributors [1]. Each component of MetS has a substantial part of heritability indicating that genetic factors may have an important influence in the pathogenesis of this syndrome. In this line, previous studies demonstrated the association of three candidate gene variants rs1799883 (*FABP2*), rs1501299 (*ADIPOQ*) and rs5065 (*ANP*), separately, with metabolic phenotypes.

The fatty acid binding protein 2 (*FABP2)* gene is located on the long arm of chromosome 4 and encodes the intestinal FABP2. The G to A transition of codon 54 results in a threonine (Thr) for alanine (Ala) substitution. Associations between *FABP2* genetic variants and different metabolic phenotypes have been reported in several studies mostly in Caucasian, American Indian or Asian populations [2–4].

Adiponectin, an adipose tissue-derived cytokine was linked to central obesity and proposed as a major contributor to MetS in addition to insulin resistance [5]. The adiponectin-encoding gene, *ADIPOQ*, is located on chromosome 3q27 within a region linked to type 2 diabetes mellitus, metabolic syndrome and coronary artery disease [6]. One of the most reported common variant, the rs1501299 (276 G>T) polymorphism, located in intron 2, was reported to be associated with MetS [7].

Several studies have demonstrated that atrial natriuretic peptide (ANP) levels are associated with obesity, metabolic syndrome and its components [8–10]. Interactions between ANP and adiponectin concentrations were also reported [11].

The genetic variant rs5065 of the *ANP* gene (chromosome 1), which introduces a stop codon that leads to the extension of the ANP peptide from 28 to 30 amino acids, is also one of the most studied *ANP* variant and has been shown to be associated to diabetic complications and cardiovascular disease [12, 13]. In the French Caribbean Island of Guadeloupe where the majority of the population is Afro-Caribbean (about 85%), there is a high prevalence of hypertension, diabetes, and obesity.

We hypothesized that each of the three variants, rs1799883 (*FABP2*), rs1501299 (*ADIPOQ*) and rs5065 (*ANP*), could be related to key underlying pathological processes of MetS and, that a genetic risk score representing their cumulative effect could be a predictor of MetS-risk in Afro-Caribbeans.

## Materials and Methods

### Study population

In a cross-sectional study conducted in the island of Guadeloupe, we studied 462 type 2 diabetic and non-diabetic patients from the Department of Diabetology and from the Health centre of the island. All participants were Afro-Caribbeans and were included in the study from 2008 to 2013. The ethnic origin was defined whether the patient defined him/herself and his/her two first-degree relatives as Afro-Caribbean. The exclusion criteria included pregnant women, patients with previous history of kidney or inflammatory disease, previous cardiovascular complications (coronary artery disease, stroke), those treated with lipid-lowering medications and those with another ethnical background. The protocol was approved by the Ethic Committee (South West—Overseas III, Bordeaux, France). All participants gave their written informed consent.

The individuals were interviewed by physicians using a standard questionnaire (S1 and S2 files).

Height and weight were measured with participants standing without shoes and lightly clothed. BMI was calculated as weight divided by height squared (kg/m2). These measurements were made by trained nurses and physicians. Blood pressure was measured according to a standardized protocol with an automatic sphygmomanometer. The retained values were the average of two or more readings. Blood samples were obtained from participants after overnight fasting. Plasma cholesterol and triglyceride concentrations were measured by enzymatic method (Boehringer-Mennheim). All usual blood tests were performed with standardized programs.

### Genotyping

Genomic DNA was extracted from peripheral white blood cells by standard methods and stored at –20°C until analysis. Genotyping of the study population was performed using a TaqMan allelic discrimination assay on an ABI PRISM 7900 HT sequence detector according to manufacturer’s instructions (Applied Biosystems, Foster City, CA, USA).

Three single-nucleotide polymorphisms (SNPs) were genotyped: rs1799883, (2445G>A) Ala54Thr of *FABP2*, rs1501299 (276G>T) of *ADIPOQ* and rs5065 (A>G, also referred as 2238T>C) of *ANP* gene.

These polymorphisms were chosen because of different data from the literature: significant associations between rs1799883 and MetS [14], association of rs1501299 with the concomitant presence of MetS and hypertension in a Taiwanese population [15], and association of rs5065 with diabetic complications and cardiovascular disease [12, 13]. In addition, previous studies in relation with cardiometabolic risk were conducted for rs1501299 and rs5065 in our Afro-Caribbean diabetic population with and without history of CAD [16, 17], and in descendants of Indian migrants living in Guadeloupe for rs1799883 [18].

### Clinical factors

Overweight was defined as BMI ≥ 25 kg/m^2^. Dyslipidaemia was defined as having at least one of the following: High-density lipoprotein-cholesterol (HDL-C) concentration < 1.04 mmol/l in men or < 1.29 mmol/l in women, triglyceride concentration ≥ 1.69 mmol/l, Low-density lipoprotein-cholesterol (LDL-C) concentration ≥ 3.40 mmol/l.

The “hypertriglyceridemic waist” phenotype (HTGW), was defined as a waist circumference ≥ 90 cm in men or ≥ 85 cm in women, along with a plasma triglyceride concentration ≥ 2.0 mmol/l in men or ≥ 1.5 mmol/l in women [19, 20].

MetS was diagnosed according to the NCEP/ATP III definition, in patients having at least three of the following five criteria: systolic blood pressure ≥ 130 and/or diastolic blood pressure ≥ 85 mmHg, abdominal obesity (waist circumference > 102 cm in men or > 88 cm in women), hypertriglyceridemia with triglyceride level ≥ 1.69 mmol/L (150 mg/dL), high-density lipoprotein-cholesterol (HDL-C) level < 1.04 mmol/L (40 mg/dL) in men and < 1.29 mmol/L (50 mg/dL) in women, fasting plasma glucose level ≥ 6.1 mmol/L (110 mg/dL) or diabetes [21].

### Statistical analysis

Data are presented as numbers (percentages) for categorical variables and as means ± standard deviations (SD) for continuous variables. The chi-squared test and ANCOVA with adjustment for age, sex and diabetes were used to test percentage and mean differences between groups, respectively.

We examined the associations between SNPs, MetS components and MetS. The logistic regression models were tested including each SNP alone with adjustment for age, sex and diabetes. Adjusted odds ratios (ORs) and 95% confidence intervals (95% CIs) were estimated for MetS and for each MetS component.

We used a genetic risk score approach to evaluate the combined effects of SNPs in relation with MetS components and MetS. The genetic risk score (GRS) was calculated by summing the number of risk alleles at each locus. ORs for metabolic syndrome according to the number of risk alleles of the 3 SNPs were also estimated.

The IBM SPSS Statistics software version 21.0 was used for data analyses. All tests were two-sided. A *P* value < 0.05 was considered to be statistically significant. A correction was applied for multi testing with a significant *P* value < 0.016.

## Results

### Characteristics of the study population

Four hundred sixty-two individuals were included in the study. Mean age was 48.9 ± 13.3 years.

Among the participants, 260 (56.3%) were women and 58 (12.6%) were people with diabetes. Overall 116 (25.1%) had MetS with 78 (19.3%) in people without diabetes vs 38 (65.5%) in people with diabetes.

The characteristics and genotype distributions of the study population according to presence or absence of MetS are shown in Table 1.

**Table 1:** Patients’ characteristics according to metabolic syndrome status.

			Metabolic Syndrome		
Characteristics			NO	YES	P
Quantitative variables*					
	**N**	**462**	**346**	**116**	
Age (year)		462	48 ± 13	53 ± 13	*< 0*.*001*
Body mass index (Kg/m^2^)		461	26 ± 5	32 ± 6	*< 0*.*001*
Waist circumference (cm)		462	87 ± 11	101 ± 11	*< 0*.*001*
Systolic blood pressure (mm Hg)		462	132 ± 18	142 ± 16	*< 0*.*001*
Diastolic blood pressure (mm Hg)		462	81 ± 11	87 ± 11	*< 0*.*001*
HDL-Cholesterol (mmol/L)		462	1.50 ± 0.47	1.17 ± 0.39	*< 0*.*001*
Triglycerides (mmol/L)		462	0.87 ± 0.91	1.54 ± 1.41	*< 0*.*001*
Fasting blood glucose (mmol/L)		462	4.87 ± 1.12	5.86 ± 2.54	*< 0*.*001*
***Qualitative variables***:					
	**N**	**462**	**346**	**116**	
Women		260	175 (50.6)	85 (73.3)	*< 0*.*001*
Men		202	171 (49.4)	31 (26.7)	
Diabetes		58	20 (5.8)	38 (32.8)	*< 0*.*001*
Overweight **		297	195 (56.4)	102 (88.7)	*< 0*.*001*
Abdominal Obesity		179	78 (22.5)	101 (87.1)	*< 0*.*001*
Hypertension		291	181 (52.3)	110 (94.8)	*< 0*.*001*
Low HDL-Cholesterol		127	58 (16.8)	69 (59.5)	*< 0*.*001*
High Triglycerides		49	21 (6.1)	28 (24.1)	*< 0*.*001*
High Fasting glucose ***		113	45 (13)	68 (58.6)	*< 0*.*001*
Hypertriglyceridemic waist		42	10 (2.9)	32 (27.6)	*< 0*.*001*
**Genetic parameters**					
***FABP2*** rs1799883	**N**	**436**	**324**	**112**	
GG		252	195 (60.2)	57 (50.9)	*0*.*086*
GA / AA		184	129 (39.8)	55 (49.1)	
***ADIPOQ*** rs1501299	**N**	**346**	**116**	**346**	
GG		170	136 (42.5)	34 (30.4)	*0*.024
GT **/** TT		262	184 (57.5)	78 (69.5)	
***NPPA*** rs5065	**N**	**430**	**323**	**107**	
AA		150	108 (33.4)	42 (39.3)	*0*.*274*
AG / GG		280	215 (66.6)	65 (60.7)	

* ANCOVA adjusted for age and sex and diabetes. Data are presented as mean ± SD or number (column percentage).

** Overweight, including obese.

*** High fasting glucose, including known diabetes. HDL: high-density lipoprotein.

The following analyses were restricted to the 415 individuals with available data for all the parameters studied including the three SNPs.

Mean levels of all the cardiovascular risk factors (except for HDL-C level) were higher in individuals with MetS than in those without MetS (*P* < 0.001 for all). Frequencies of overweight, HTGW and all the MetS components were higher in individuals with MetS than in those without MetS (*P* < 0.001 for all).

The genotype distributions in the overall study population were within the Hardy—Weinberg equilibrium for rs1799883 G>A (57.8% GG, 34.9% GA, 7.3% AA; *P* = 0.17), rs1501299 G> T (39.4% GG, 46.5% GT, 14.1% TT; *P* = 0.90) and rs5065 A>G (34.9% AA, 48.6% AG, 16.5% GG; *P* = 0.90).

The frequencies of *ADIPOQ-* rs1501299 (GT/TT) and of *FABP2-* rs1799883 (GA/AA) minor allele carriers in individuals with and without MetS were 69.5% *vs* 57.5% (*P* = 0.024) and 49.1% *vs* 38.9% (*P* = 0.086), respectively.

### Logistic regressions of metabolic syndrome components, metabolic syndrome overweight and HTGW according to genotypes

In Table 2 are presented the adjusted ORs for risk of hypertension, abdominal obesity, Low-HDL cholesterol levels, high triglycerides levels, high fasting blood glucose levels and MetS according to the three SNP genotypes. The regressions were performed separately for each SNP after adjustments for age, sex and diabetes.

**Table 2:** Logistic regressions of metabolic syndrome components and metabolic syndrome for rs1799883 (*FABP2*), rs1501299 (*ADIPOQ*) and rs5065 (*ANP*) gene polymorphisms.

		Hypertension		Abdominal obesity		Low HDL-Cholesterol		High Triglycerides		High fasting blood Glucose		Metabolic syndrome	
	N												
	415	OR (95% CI)	*P*	OR (95% CI)	*P*	OR (95% CI)	*P*	OR (95% CI)	*P*	OR (95% CI)	*P*	OR (95% CI)	*P*
**rs1799883 *(FABP2*)**													
**GG**	**244**	1		1		1		1		1		1	
**GA / AA**	**171**	1.50 (0.97–2.33)	*0*.*068*	1.67 (1.04–2.66)	***0*.*033***	1.28 (0.82–1.99)	*0*.*277*	2.22 (1.18–4.19)	***0*.*014***	0.83 (0.48–1.43)	*0*.*495*	1.59 (0.97–2.62)	*0*.*068*
**rs1501299 *(ADIPOQ*)**													
**GG**	**166**	1		1		1		1		1		1	
**GT / TT**	**249**	1.11 (0.71–1.71)	*0*.*641*	1.28 (0.80–2.04)	*0*.*311*	1.11 (0.71–1.75)	*0*.*647*	1.49 (0.77–1.86)	*0*.*235*	1.22 (0.74–2.01)	*0*.*434*	1.80 (1.07–3.05)	***0*.*028***
**rs5065 *(ANP*)**													
**AG / GG**	**270**	1		1		1		1		1		1	
**AA**	**145**	1.20 (0.77–1.87)	*0*.*431*	1.32 (0.82–2.13)	*0*.*249*	1.49 (0.95–2.35)	*0*.*081*	1.77 (0.94–3.31)	*0*.*075*	0.91 (0.52–1.60)	*0*.*749*	1.24 (0.74–2.06)	*0*.*413*

ORs were calculated separately for each SNP after adjusting for age, gender and diabetes

In a dominant model, the rare allele of rs1799883 (*FABP2)* (GA/AA vs GG) was associated with prevalence of high triglycerides levels (OR 2.22; *P* = 0.014) but not with prevalence of abdominal obesity (OR 1.67; *P* = 0.033) and MetS (OR 1.59; *P* = 0.068).

Carriers of the minor allele of rs1799883 had also an increased risk of HTGW phenotype compared to GG homozygotes (*P* = 0.014), Table 3.

**Table 3:** Logistic regressions of overweight and hypertriglyceridemic waist for rs1799883 (*FABP2*), rs1501299 (*ADIPOQ*) and rs5065 (*NPPA*) gene polymorphisms.

		Overweight		Hypertriglyceridemic waist	
	N	OR (95% CI)	*P*	OR (95% CI)	*P*
	**415**				
***FABP2*** rs1799883					
GG	**244**	1		1	
GA / AA	**171**	1.53 (1.01–2.33)	*0*.*049*	2.32 (1.19–4.52)	*0*.*014*
***ADIPOQ*** rs1501299					
GG	**166**	1		1	
GT / TT	**249**	1.01 (0.47–1.53)	*0*.*961*	2.19 (1.04–4.64)	*0*.*040*
***ANP*** rs5065					
AG / GG	**270**	1		1	
AA	**145**	1.14 (0.74–1.75)	*0*.*556*	1.27 (0.65–2.47)	*0*.*477*

ORs were calculated separately for each SNP after adjusting for age, gender and diabetes

In a dominant model, the OR of MetS and HTGW phenotype for the rare allele of rs1501299 (*ADIPOQ)* (GT/TT vs GG) were 1.80 (*P* = 0.028) and 2.19 (*P* = 0.040) respectively, Table 3.

The ORs of Low HDL-C and high triglycerides levels for the most frequent allele of rs5065 (*ANP)* in a recessive model (AA vs AG/GG) were 1.49 (*P* = 0.081) and 1.77 (*P* = 0.075), respectively, Table 2.

Table 4 shows the adjusted ORs of MetS for age, gender, diabetes, rs1799883 (*FABP2*), rs1501299 (*ADIPOQ*) and rs5065 (*ANP*). Age (OR 1.04; *P* = 0.001), gender (OR 2.95; *P* < 0.001) and diabetes (OR 5.03; *P* < 0.001) were associated with an increased risk of MetS. The ORs of MetS for carrying the rare allele (GA + AA) of rs1799883 (*P* = 0.087), the rare allele (GT + TT) of rs1501299 (*P* = 0.035) or the frequent allele AA of rs5065 (*P* = 0.549) did not reach significance.

**Table 4:** Logistic regression of metabolic syndrome with age, gender, diabetes and the three gene polymorphisms (rs1799883, rs1501299 and rs5065) as covariates.

		OR (95% CI)	*P*
Age (y)	415	1.04 (1.02–1.06)	*0*.*001*
Gender			
Male	186	1	
Female	229	2.98 (1.75–5.08)	*<0*.*001*
Diabetes			
No	372	1	
Yes	43	5.03 (2.33–10.9)	*<0*.*001*
*FABP2* rs1799883			
GG	244	1	
GA / AA	171	1.55 (0.94–2.57)	*0*.*087*
*ADIPOQ* rs1501299			
GG	166	1	
GT **/** TT	249	1.77 (1.04–3.00)	*0*.*035*
*ANP* rs5065			
AG / GG	270	1	
AA	145	1.17 (0.70–1.96)	*0*.*549*

Adjusted ORs were calculated for the six covariates included concomitantly in the model.

### Relationship between GRS and metabolic syndrome

The risk alleles for MetS were A for rs1799883, T for rs1501299 and A for rs5065. The GRS was calculated for each individual by summing the number of risk allele at each locus (0 if absence of risk allele, 1 if heterozygote, 2 if homozygote for the risk allele). The mean GRS was significantly higher in patients with high triglyceride levels (2.76 *vs* 2.36 respectively; *P* = 0.036) and in those with MetS (2.67 *vs* 2.32 respectively; *P* = 0.021) (Table 5).

**Table 5:** Mean genetic risk score, in 415 individuals, according to presence or absence of metabolic syndrome or metabolic syndrome components.

	Metabolic syndrome or metabolic syndrome components				
	YES		NO		*P*
	N	GRS ± SD	N	GRS ± SD	
**Metabolic syndrome components**					
Abdominal Obesity	157	2.53 ± 1.29	258	2.33 ± 1.15	*0*.*141*
Hypertension	262	2.46 ± 1.21	153	2.29 ± 1.20	*0*.*187*
Low HDL-Cholesterol	118	2.49 ± 1.25	297	2.37 ± 1.19	*0*.*368*
High Triglycerides	47	2.76 ± 1.06	368	2.36 ± 1.22	*0*.*036*
High FBG or diabetes	105	2.47 ± 1.29	310	2.38 ± 1.18	*0*.*617*
**Metabolic syndrome**	102	2.67 ± 1.30	313	2.32 ± 1.16	*0*.*021*

Data are mean ± SD.

ANCOVA adjusted for age, gender and diabetes

**Fig 1:**
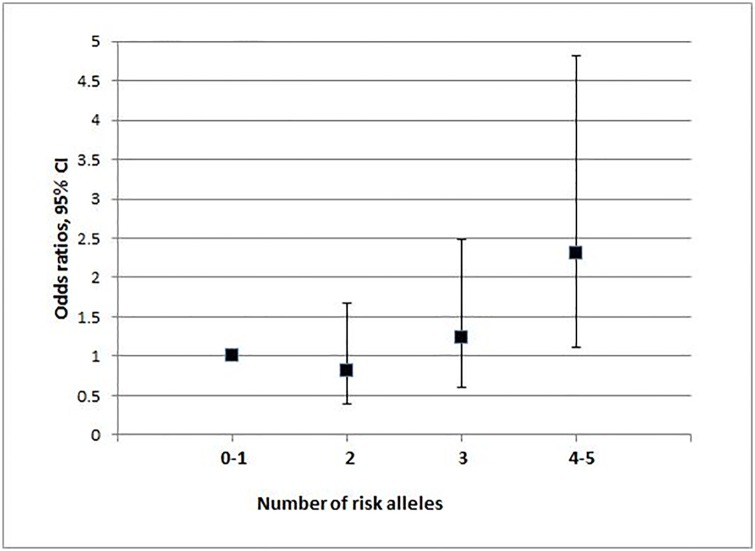
Odds ratios for metabolic syndrome according to the number of risk alleles of the 3 SNPs: rs1799883 (Ala54Thr), rs1501299 (276 G>T), rs5065 (2238 T>C). Number of risk alleles 0–1: reference group. For 2 risk alleles: *P* = 0.57. For 3 risk alleles: *P* = 0.50. For 4–5 risk alleles: *P* = 0.025.

After adjustment for age, gender and diabetes and taking patients with 0–1 risk allele as the reference group (24.3%), the odds ratio (OR) of MetS for patients with 4–5 risk alleles (18.8%) was 2.31 (95% CI 1.11–4.81; *P* = 0.025) (Fig 1).

## Discussion

In the present study we examined the association of rs1501299 (*ADIPOQ)*, rs1799883 (*FABP2)* and rs5065 (*ANP)* variants with MetS-risk in an Afro-Caribbean population. The main finding of our study is that a genetic risk score summarizing the influence of these 3 SNPs, was associated with metabolic syndrome. Our results are in favour of a genetic contribution leading to the development of MetS in this population.

There are strong evidences that ANP, FABP2 and adiponectin play an important role in key metabolic processes and especially in lipids metabolism. The three SNPs investigated in this study are among the most studied variants of their respective genes and two of them (rs5065-*ANP* and rs1799883-*FABP*2) affect directly the function of the cognate protein. These SNPs were also selected based on prior knowledge from studies conducted in our population.

The sample from this multi-ethnic population included only people of Afro-Caribbean origin determined if the patient defined him/herself and his/her two first-degree relatives as Afro-Caribbean. We assumed that the combination of these inclusions criteria allowed the selection of a homogeneous and representative sample of the Afro-Caribbean population. We have previously shown in a sample selected by these criteria, from our population, that the distribution of SNP-allele frequencies for 19 SNPs was very close to that of the Afro-Caribbean from Barbados published in 1000 genome [22].

### Fatty acid binding protein 2, rs1799883 and metabolic syndrome

*FABP2* Ala54Thr polymorphism (rs1799883) was significantly associated with hypertriglyceridemia (OR = 2.22; *P* = 0.014). A significant association was also noted with hypertriglyceridemic waist phenotype which is known as an optimal screening tool to identify subjects with MetS and increased risk of cardiovascular disease [19].

Fatty acid-binding proteins (FABPs) are members of the super family of small intracellular lipid-binding proteins and intestinal FABP (I-FABP or FABP2) is one of nine different FABPs. FABP2 has a high affinity for saturated and unsaturated long-chain fatty acids, and is widely understood to be involved in the absorption, growth and transport of dietary fatty acids and to increase fat oxidation, which has been shown to reduce insulin action [23].

Among the factors involved in MetS, genetic polymorphisms are often implicated, especially those responsible for metabolism and transport of lipids [24], regulation of blood pressure and regulation of glucose metabolism.

It has been reported that the Ala54Thr polymorphism of *FABP2* (rs1799883), has an important effect on postprandial lipids as the threonine-containing protein has a twofold greater affinity for the long chain fatty acids than the alanine-containing protein [23, 25]. As a result, higher fasting plasma levels of cholesterol and TG (especially in postprandial stage) were found in subjects carrying the Thr54 mutation [26]. This excessive level of fatty acids (associated with the Thr54) and their preferential use as a source of energy by skeletal muscle rather than glucose, contribute to an increase in glucose levels, higher basal and glucose-stimulated insulin levels and higher degree of insulin resistance [2, 14, 23]. Significant associations between the FABP2 Ala54Thr polymorphism and MetS components or MetS were reported in some studies [3, 14, 26] whereas some others found no association [27].

In a population of Asian Indian descent living in Guadeloupe, Boullu-Sanchiz et al. reported a significant relation between the FABP2 Ala54Thr polymorphism and type 2 diabetes, that seemed to be related to the metabolic insulin resistance syndrome [18]. Recently a meta-analysis by Liu et *al*, revealed significant associations for metabolic syndrome and type 2 diabetes, suggesting the implication of *FABP2* gene in the pathogenesis of MetS [14]. In our present study, this association with MetS was not significant (OR 1.59; *P* = 0.068).

### Adiponectin, rs1501299 and metabolic syndrome

Low plasma adiponectin levels have been associated with insulin resistance, and increased risk of type 2 diabetes [28, 29]. In the present study, the risk ratio of MetS for carriers of the T allele (GT/TT) of rs1501299 was 1.80; *P* = 0.028. This lack of significance may be due to a lack of power given the size of our study population. In fact, for an identical OR of 1.80 (Table 2), with a 80% study power, a type 1 error of 5% and a significant p value < 0.016, we should have a sample size of at least 760 subjects (including 190 subjects with MetS).

Both T and G alleles of rs1501299 (*ADIPOQ)* were reported to be associated with metabolic phenotypes or with adiponectin concentrations. In a meta-analysis for association between *ADIPOQ* rs1501299 and MetS, most of the studies showed trends of elevated OR for the G allele [7]. But, as in our study population, others showed trends of elevated OR for the T allele [30, 31]. In 1438 Taiwanese subjects, the OR for MetS for GT/TT genotypes (vs *GG* genotype) was 1.30; *P* = 0.015 [31]. Ronconi et *al* found that the rs1501299 TT genotype was associated with a significantly worse metabolic profile and a higher risk for MetS (OR = 1.5 in patients with primary aldosteronism and OR = 1.3 in those with essential hypertension) [30]. These inconsistent results are probably due to different diagnostic criteria for MetS components or MetS but also to ethnic variations and to gene-environment interactions. The rs1501299 (*ADIPOQ)* has been associated with adiponectin levels in Genome-Wide Association Studies (GWAS) [32, 33] but, the association of rs1501299 with metabolic disorders is not systematically related to differences in plasma adiponectin [34, 35]. The effect of the genetic variant may be independent of the adiponectin circulating levels [36].

### ANP, rs5065 and metabolic syndrome

In our study, the associations between the *ANP* gene variant rs5065 and MetS or MetS components were not significant. Nevertheless, there is increasing evidence for a key role for natriuretic peptides, mainly ANP, in human metabolism and cardio-metabolic disorders. It has been demonstrated that ANP can enhance lipolysis, lipid mobilization and oxidation [37, 38] and can also act indirectly by stimulating the release of adiponectin from adipose tissue [11]. In addition ANP exhibits anti-inflammatory effects which can be of physio-pathological importance in the low chronic inflammation state of adipose tissues, involved in insulin-resistance and cardiovascular complications especially in obese subjects [39].

The *ANP* gene variant rs5065 has been associated with hypertension, myocardial infarction and stroke [40] but to our knowledge, no study had focused on the association with lipids levels. Some studies indicate that the minor allele of rs5065 variant lead to an altered ANP causing increased permeability and, reduced viability, proliferation and migration, in endothelial cells, that could explain the association of this variant with cardiovascular disease [41, 42]. However, the potential effects of rs5065 on the metabolic properties of the cognate peptide have not been studied and it is still unclear whether rs5065 is associated with a modification of ANP levels or not [43]. Therefore, the functional significance of rs5065 on lipid parameters and metabolic syndrome remains to be elucidated by further investigations.

### Association of the GRS with hypertriglyceridemia and metabolic syndrome

The GRS summarizing the influence of the 3 SNPs was associated with metabolic syndrome. Patients with 4–5 risk alleles have nearly 2.5-fold higher risk of MetS compared to those with no or 1 risk allele. In addition, the GRS was significantly higher in subjects having high triglyceride levels or MetS than in the others.

Taken together, our data support the idea that fatty acids and triglycerides regulation/dysregulation plays a crucial role in the development of MetS and reinforce the evidence for a polygenic component in this syndrome. In a systematic review on the most studied SNP-MetS associations, the authors found an association with MetS for SNPs, mostly located in genes involved in lipid metabolism like FTO rs9939609, TCF7L2 rs7903146, APOA5 rs662799, APOC3 rs2854117, IL6 rs1800795, and CETP rs708272 [24].

Increased circulating triglycerides are associated with an atherogenic lipid profile and insulin resistance, resulting in an increased risk for metabolic syndrome diabetes and cardiovascular disease, especially in obese subjects [44]. Hypertriglyceridemia may be the consequence of enhanced liberation of fatty acids from excessive visceral adipose tissue leading to enhanced hepatic synthesis of VLDL [45, 46]. The association of GRS with MetS and hypertriglyceridemia found in our study could reflect the cumulative influence and the interaction of *ANP*, *FABP2* and *ADIPOQ* genes that are implicated in fatty acid metabolism in different key tissues and cells such as enterocytes, adipocytes, skeletal muscle and liver.

Limitations of this study include its relatively small sample size. In addition, environmental risk factors for MetS such as exercise behaviour or dietary patterns were not taken into account even if they are moderately heritable. However, our study design excluding patients treated with lipid-lowering medications and with previous cardiovascular complications provides a reliable assessment of the association between SNPs, hypertriglyceridemia and MetS in this sample of Afro-Caribbean subjects.

In conclusion, the results of this study indicate that *FABP2*, *ANP* and *ADIPOQ* gene variants are associated with metabolic syndrome or its components in Afro-Caribbeans and suggest a cumulative effect of these variants on the risk of metabolic syndrome and hypertriglyceridemia. Further studies are needed in a large population and other ethnic groups to confirm theses genetic associations and to investigate the underlying physiopathological mechanisms.

## Supporting Information

S1 File Questionnaire in English. (PDF)

S2 File Questionnaire in French. (PDF)
