# Supplementary material for: Gene Polymorphisms of FABP2, ADIPOQ and ANP and Risk of Hypertriglyceridemia and Metabolic Syndrome in Afro-Caribbeans
Source: PLoS One. 2016 Sep 29;11(9):e0163421. doi: 10.1371/journal.pone.0163421 (PMC5042446; doi:10.1371/journal.pone.0163421)
Supplement: S1 File — (PDF) [file pone.0163421.s001.pdf]

Questionnaire in English

Cardio Metabolic Risk

Date: /__ _/_ __/____ /

Diabetes: ☐yes ☐no if yes T2D : ☐yes ☐no
Diabetes vintage: /______/ year

<u>Demographic Parameters</u>
Number: /__________/
Group: /___/ (Case =1; Control = 0) (case = History of CAD or Stroke)

Gender: ☐Male ☐Female (1=M; 2=F)
Birth date: /_ __/_ __/__ ___/
Place of birth: __________ ____________
ZIP code of residence: ________________

<u>History related to cardiovascular and arterial diseases</u>.

Family history

Fam hist for hypertension: ☐yes ☐no

Fam hist for diabetes: ☐yes ☐no

Personal

Hypertension: ☐yes ☐no

Stroke: ☐yes ☐no

CAD: ☐yes ☐no

Myocardial Infarction : ☐yes ☐no

Angina: ☐yes ☐no

Lower limbs arteriopathy: ☐yes ☐no if yes, Amputation : ☐yes ☐no

<u>Mode de vie</u>

Tobacco: ☐yes ☐no (yes = current smoker or stop for less than 10 years, no = others)

Alcohol: ☐yes ☐no

If yes: wine ☐yes ☐no; rum:☐yes ☐no; nbr glasses / day / __ /

Sports: ☐yes ☐no; if yes nbr hours weekly / __ /

Current treatment

Antihypertensive: ☐yes ☐no

ACE: ☐yes ☐no Calcium blocker : ☐yes ☐no

Beta blocker: ☐yes ☐no other Tt : ☐yes ☐no if yes : ________________

Anti-diabetic Tt: ☐yes ☐no

If yes ☐OAD ☐Insulin ☐both

Hypolipemic Tt: ☐yes ☐no

Antiplatelet agents: ☐yes ☐no

Clinical data

Weight:/______/kg Height: /______/m Waist circumference /______ / cm

Electrocardiogram

ECG: ☐normal ☐abnormalities

If abnormal: ischemic ☐yes ☐no

Biological data

C-RP: /___/ mg/L
Hemoglobin: /___/ g/dl Hématocrite: /___/ %
Creatinine: /__ _/ µmol/L
Total Cholesterol: /__ _/ mmol/L
HDL-Cholesterol: /__ _/ mmol/L
LDL-Cholesterol: /_ __/ mmol/L
Triglycerides: / __ _/ mmol/L
FBG: /___ __/ mmol/L
