# Supplementary material for: Gene Polymorphisms of FABP2, ADIPOQ and ANP and Risk of Hypertriglyceridemia and Metabolic Syndrome in Afro-Caribbeans
Source: PLoS One. 2016 Sep 29;11(9):e0163421. doi: 10.1371/journal.pone.0163421 (PMC5042446; doi:10.1371/journal.pone.0163421)
Supplement: S2 File — (PDF) [file pone.0163421.s002.pdf]

Questionnaire in French

Risque Cardio Métabolique

Date : /__ _/_ __/____ _/

Diabète ☐ oui ☐ non Si oui DT2 : ☐ oui ☐ non
Ancienneté du diabète : /______/ ans

<u>Paramètres démographiques</u>
Num : /__________/
Groupe : /___/ (Cas =1 Témoin = 0) (cas = ATCD de coronaropathie ou AVC)

Sexe : ☐ Masculin ☐ Féminin (1=M, 2=F)
Date de naissance : /_ __/_ __/__ ___/
Lieu de naissance : ___________
Code postal de résidence : ___________

<u>Antécédents relatifs aux pathologies cardiovasculaires et artérielles</u>.
<u>Familiaux</u>

Familiaux d'HTA : ☐ oui ☐ non

Familiaux de diabète : ☐ oui ☐ non
<u>Personnels</u>

Hypertension : ☐ oui ☐ non

Personnel AVC : ☐ oui ☐ non

Coronaropathie : ☐ oui ☐ non

Infarctus : ☐ oui ☐ non

Angor : ☐ oui ☐ non

Artériopathie : ☐ oui ☐ non si oui, Amputation : ☐ oui ☐ non

Mode de vie

Tabac : ☐ oui ☐ non (oui=fumeur actuel ou arrêt depuis moins de 10 ans, non =les autres)

Alcool : ☐ oui ☐ non Type vin ☐ oui ☐ non rhum ☐ oui ☐ non
si oui nbr verres/jour /__/

Pratique sportive : ☐ oui ☐ non si oui nbr heures hebdomadaires /__/

<u>Traitement en cours</u>

Hypotenseur : ☐ oui ☐ non

IEC : ☐ oui ☐ non Inhibiteurs calciques : ☐ oui ☐ non

Bétabloquants : ☐ oui ☐ non Autres Tt : ☐ oui ☐ non si oui type Tt :

Antidiabétiques :

☐ ADO ☐ insuline ☐ les deux

Hypolipémiant: ☐ oui ☐ non

Antiagrégants plaquettaires : ☐ oui ☐ non

<u>Données cliniques</u>

Poids : /____/ kg Taille : /____/ m Tour de taille /____ / cm

<u>Electrocardiogramme</u>

ECG : ☐ normal ☐ anomalies

Si anomalie : ischémiques ☐ oui ☐ non

<u>Données biologiques</u>

CRP : /___/ mg/L
Hémoglobine : /___/ g/dl Hématocrite : /___/ %
Urée : /___/ mmol/L Créatinine : /__ _/ µmol/L
Cholestérol total : /__ _/ mmol/L
HDL cholestérol : /__ _/ mmol/L
LDL cholesterol : /_ __/ mmol/L
Triglycérides : / __ _/ mmol/L
Glycémie : /_ __/ mmol/L
